# Memoir of a Gentleman Born Blind and Successfully Operated on in the 18th Year of His Age

**Published:** 1842-04

**Authors:** 


*Memoir of a Gentleman born blind and successfully operated on in the* 18*th year of his age.* By Dr. Franz.—At the birth of this young gentleman (the son of a physician) the eyes were found to present a twofold defect of organization. Both eyes were turned inwards to a great extent—and cataract existed in both. Towards the end of the second year, keratonyxis was performed on the *right* eye, which was followed by iritis and wasting of the eye-ball. Within the next four years, two similar operations were performed on the *left* eye, without any success, but with no destruction of the eye. The color of the opacity, at length, became of a clearer white and some faint perception of a strong light was experienced by the boy.

Into the long and minute description of the state of this gentleman's eyes in his 18th year (1840) we cannot go. It appeared that the *right* eye was completely amaurotic, and the *left*, which had become atrophied, was the only one considered fit for an operation. The following were the steps taken.

"On the 10th of July 1840, in the presence of Dr. Swaine and with the kind assistance of Messrs. F. Fowke and F. Steinhaeuser, I made an incision in the cornea upwards, and introducing a pair of fine curved forceps, armed with teeth, into the posterior chamber, I seized the anterior wall of the capsule by passing one of the blades of the forceps into its small aperture, and attempted, by pulling it slowly, to separate it from its adhesion with the uvea and its peripheral connection, in which I succeeded without producing a prolapsus of the vitreous body, or tearing the capsule, which I now removed. After this proceeding, a large piece of the lens of an opaque color, probably the nucleus, presented itself in the pupil, which was easily removed from the eye by means of Daviel's spoon; the pupillary aperture then appeared perfectly clear and black. The patient was now turned with his back to the light, for the purpose of trying a few experiments as to his sight, but from these I was obliged to desist on account of the pain which the light produced in the organ. Both eyes were then closed with narrow strips of court-plaster, and the patient carried to bed. Venesection, local bleeding, fomentations of iced water, continued without intermission for about forty-eight hours, together with the scrupulous observance of the most severe regimen, barely succeeded in keeping down the inflammation, the effects of which in this case, where but one eye offered hope, were much to be dreaded, if it should surpass that degree which was necessary for the healing of the wound in the cornea. This process went on and terminated so favorably, that the cicatrix, situated close to the slerotica, is now scarcely visible. The patient suffered from muscæ volitantes and from a considerable intolerance of light, pain being produced by even a mild degree of light falling on the closed lids. The muscæ volitantes were greatly mitigated, and the intolerance of light ceased after the lapse of a few weeks, by the use of proper pharmaceutical remedies, by local bleeding, change of air, &c., and the employment of the ophthalmic fountain of Professor Jungken, which I have fully described in the Medical Gazette, vol. xxvii, p. 444. To promote the development of the power of vision, the use of the fountain was continued twice daily, with Pyrmont water, and latterly with simple spring-water, for the space of three months, when it was discontinued, as it began to irritate the eye."

On opening the eye on the third day, he perceived a blaze of light, and all objects confused and in motion. He could not distinguish any object. The pain forced him quickly to close the eye. Gradual exposure of the eye to light habituated the organ to its stimulus; and when the vision became tolerably distinct, all objects appeared so near to him that he was afraid of coming in contact with them, so that he was constantly correcting the sense of sight by that of touch.

On the 21st of September, 1840, Dr. F. operated on both eyes for the congenital strabismus. The operation was so successful, that the gentleman's personal appearance was much improved. In November he was able to read the names over the shop windows, and to tell the time, to a minute, by the St. Paul's clock. The tide of human existence, however, in the streets, so confused and confounded him, that at last he could see nothing, By the spring of 1841, the sight was much improved—and improving. The case, altogether, is very creditable to Dr, Franz, as well as interesting to the profession and the public. The paper is published in the Philosophical Transactions for 1841, Part 1.—*Med. Chir. Rev.*

